# Laboratory Investigations on the Potential Efficacy of Biological Control Agents on Two Thrips Species, Onion Thrips (*Thrips tabaci* Lindeman) and Western Flower Thrips (*Frankliniella occidentalis* (Pergande))

**DOI:** 10.3390/insects15060400

**Published:** 2024-05-30

**Authors:** Ashley Summerfield, Rosemarije Buitenhuis, Sarah Jandricic, Cynthia D. Scott-Dupree

**Affiliations:** 1 Vineland Research and Innovation Centre, Vineland Station, ON L0R 2E0, Canada; rose.buitenhuis@vinelandresearch.com; 2 Ontario Ministry of Food, Agriculture, and Rural Affairs, Vineland Station, ON L0R 2E0, Canada; sarah.jandricic@ontario.ca; 3 School of Environmental Sciences, University of Guelph, Guelph, ON N1G 2W1, Canada; cscottdu@uoguelph.ca

**Keywords:** thrips, greenhouse IPM, biological control, integrated pest management, predatory mites, *Orius insidiosus*, entomopathogenic nematodes, entomopathogenic fungi

## Abstract

**Simple Summary:**

Thrips are among the most damaging insect pests affecting greenhouse horticulture crops. Research on biological control of these pests has focused on the dominant species, western flower thrips (WFT). However, a second species, onion thrips (OT), has become more prevalent in greenhouse ornamentals in Ontario, and biocontrol strategies for WFT do not control OT sufficiently to prevent crop losses. Although thrips’ natural enemies have been tested against OT alone, there are few studies examining how effective they are for OT compared to WFT. We conducted several laboratory trials examining the relative efficacy of several natural enemies typically used in thrips biocontrol, including predators, parasitic nematodes, and a fungal-based biopesticide. All of the natural enemies tested were at least as effective at killing OT as they were for WFT in the laboratory, indicating that they all have the potential to manage both species equally well. Possible explanations why this potential is not realized in commercial greenhouse operations are explored.

**Abstract:**

Thrips biocontrol research in greenhouse crops has focused primarily on western flower thrips (WFT; *Frankliniella occidentalis*). However, recent outbreaks of onion thrips (OT; *Thrips tabaci*) in Ontario, Canada, demonstrate that biocontrol-based IPM programs for WFT do not control OT sufficiently to prevent crop losses. A lack of comparative studies makes it difficult to determine which program components for WFT are failing for OT. We conducted several laboratory trials examining the extent to which commercial biocontrol products kill OT compared to WFT. These included phytoseiid mites (*Amblyseius swirskii*, *Neoseiulus cucumeris*, *Amblydromalus limonicus*, *Iphiseius degenerans*), a large generalist predator (*Orius insidiosus*), an entomopathogenic fungus *(Beauveria bassiana* strain GHA), and entomopathogenic nematodes (*Steinernema feltiae*, *S. carpocapsae*, *Heterorhabditis bacteriophora*). In no-choice trials, *A. swirskii* and *O. insidiosus* consumed more OT than WFT (first instars and adults, respectively). In choice trials, *A. swirskii*, *N. cucumeris*, and *O. insidiosus* consumed more OT than WFT. *Steinernema feltiae* caused higher mortality in OT than WFT. There was no difference in mortality between thrips species exposed to other biocontrol agents. This suggests available tools have the potential to manage OT as well as WFT. Possible explanations why this potential is not realized in commercial settings are explored.

## 1. Introduction

Integrated pest management (IPM) in greenhouse ornamentals typically requires the simultaneous use of multiple biological control agents (BCAs) for a single pest to achieve the low pest levels required of plants grown for their aesthetic appeal [1,2]. Predatory phytoseiid mites are usually the first line of defense against thrips [1,3], due to both their efficacy and minimal cost per predator. Entomopathogenic fungi and nematodes are often used in conjunction to augment control [4,5]. Research on the efficacy of these biocontrol agents in greenhouse crops in temperate climates has largely focused on the primary pest species, western flower thrips (WFT; *Frankliniella occidentalis* (Pergande)). As a consequence, thrips IPM strategies have been optimized for this species. However, another thrips species, onion thrips (OT; *Thrips tabaci* Lindeman), is becoming more prevalent in greenhouse ornamentals in Ontario, Canada [6], which represents a large ornamental production region in North America [7].

Ontario growers with mixed populations of OT and WFT previously operated under the assumption that thrips biocontrol strategies should be equally effective at suppressing both species. However, adequate control of OT is not being achieved with the biocontrol program that typically works well for WFT-only populations (SJ, personal observation). A likely explanation is that one or more components of the thrips IPM program are not equally effective for both thrips species. Since greenhouse IPM strategies implement multiple components, it remains unclear which of these components is falling short.

*Neoseiulus cucumeris* (Oudemans) and *Amblyseius swirskii* (Athias-Henriot) are the two mite species most commonly used by floriculture growers in Ontario (S.J., personal observation). The predatory hemipteran, *Orius insidiosus* (Say), is occasionally used to provide additional support to predatory mites in some crops [8]. Published research confirms that *A. swirskii*, *N. cucumeris*, and *O. insidiosus* all consume OT [9,10,11,12].

However, with no studies directly comparing predation rates between OT and WFT, we cannot conclude whether these predators consume equal numbers of both thrips species. As these two thrips species are typically found in mixed populations [6], prey preference could play an important role in how effectively predators manage them. Previous research has demonstrated that phytoseiid mites and *Orius* spp. do exhibit prey preferences [13,14,15,16,17,18]. However, only one study has looked at prey preference between different thrips species [19]. That study, examining the preference and predation rate of *Orius laevigatus* on WFT and *Frankliniella bispinosa* Morgan, confirmed that predators can exhibit prey preference even between congeneric species.

The prevalence of pesticide-resistant OT in crops such as onions and cabbage has generated interest in alternative strategies that are practical for use in outdoor agricultural systems [20,21,22]. Consequently, more extensive research has been published on the use of entomopathogens for OT than there has been for augmentative releases of predatory BCAs against this pest. However, much of the research on entomopathogenic fungi (EPF) and nematodes (EPN) has been conducted on fungal and nematode strains cultivated from wild hosts, not commercial products. The variability in efficacy between different strains has been well documented for both EPF [21,23,24] and EPN [25,26,27], so testing specific commercially-available strains is imperative. Further, only one study has directly compared the relative efficacy of EPF for managing both OT and WFT [28], and no comparative studies have been published for EPN.

It also is uncertain if previous laboratory results will translate into effective control in greenhouse crops. This is especially true for EPN that have been studied using mineral soils or filter paper as substrates. Floriculture crops are grown in soilless media, which differ substantially from mineral soils in properties such as moisture retention, particle size, porosity, and water potential [29]. These properties affect the movement and host finding capacity of EPN, and therefore their efficacy as biocontrol agents [30,31,32]. For this reason, it is essential to study EPN in the substrate in which they will be used.

As it can take several years for new biocontrol agents to be developed for commercial use, it is necessary to find non-pesticide solutions among existing products for OT, if possible, to limit disruptions of successful biocontrol-based programs for other greenhouse pests in Ontario [33]. There may be potential solutions among some underutilized BCAs that are already available on the Canadian market. Two other thrips predators that are commercially available, but used less frequently due to cost, are *Ambydromalus limonicus* (Garman and McGregor) and *Iphiseius degenerans* (Berlese). On cucumber plants, *A. limonicus* has a higher predation rate on WFT than *N. cucumeris* and *A. swirskii* [34,35], and *I. degenerans* has a higher predation rate than *N. cucumeris* on some host plants [36], so both could be good candidates for OT control. Of the commercially available EPNs, *Steinernema feltiae* (Filipjev) is the species typically used in greenhouse crops [5,37]. Two other species, *S. carpocapsae* (Weiser) and *Heterorhabditis bacteriophora* Poinar, are typically recommended for other soil-dwelling pests such as beetle larvae and caterpillars [38]. However, numerous studies have demonstrated they can also infect and kill thrips [20,25,39,40], so their potential efficacy against OT should be explored.

Overall, the goals of this research were to (1) determine the relative predation rate and prey preference of the predators *A. swirskii*, *N. cucumeris*, *I. degenerans*, *A. limonicus,* and *Orius insidiosus* against OT and WFT in the laboratory in no-choice and choice assays; (2) determine the relative efficacy of commercially produced *S. feltiae*, *S. carpocapsae*, and *Heterorhabditis bacteriophora* against OT and WFT pupating in soilless media; and (3) determine whether OT and WFT differ in their susceptibility to a commercially available EPF, *Beauveria bassiana* GHA. Laboratory studies such as these are an important first step in elucidating why the differential control of greenhouse pests may occur. Based on our results, possible explanations why the potential of biocontrol agents for OT is not realized in commercial greenhouse operations are explored, and future research avenues are proposed.

## 2. Materials and Methods

### 2.1. Insects and Biological Control Agents

#### 2.1.1. Thrips

An OT colony was established in 2019 from individuals collected from chrysanthemums (*Chrysanthemum indicum*) at a commercial greenhouse and marigolds (*Tagetes patula*) at the Vineland Research & Innovation Centre (‘Vineland’) in Vineland, Ontario, and was supplemented with adults collected from marigolds at Vineland in 2020 and 2021. The colony was subsequently reared on miniature cucumber fruits (*Cucumis sativus*) [41] and cabbage leaves (*Brassica oleracea*). A WFT colony was established in 2015 from individuals collected from chrysanthemum at a commercial greenhouse, and supplemented annually with adults collected from roses (*Rosa*, shrub rose breeding collection) at Vineland. The main WFT colony was reared on a combination of marigold plants (*T. patula* ‘Bonanza yellow’), and bean plants (*Phaseolus vulgaris* ‘California red kidney’). Additional WFT colonies were reared on miniature cucumber fruits for use in these trials. Both thrips colonies were reared in growth chambers (24 °C, 60%RH, 16 h light).

#### 2.1.2. Predators

Predatory mites *A. swirskii*, *I. degenerans*, *A. limonicus*, and *N. cucumeris*, and the predatory bug, *O. insidiosus*, were obtained from Biobest Canada Ltd. (Leamington, ON, Canada). New predators were ordered for each trial and temporarily housed in a growth chamber (25 °C, 70%RH, 16 h light) for up to 2 weeks. The mites and carrier materials (vermiculite or bran) were transferred to vented plastic containers (22 × 22 × 10 cm) prior to the assays and placed on top of sponges in a larger tray filled with water with 1–2 mL of unscented dish soap to prevent escape. The mites were provided with *Ephestia kuehniella* eggs (Beneficial Insectary, Redding, CA, USA) and cattail pollen (Nutrimite^®^, Biobest Canada Ltd., Leamington, ON, Canada) adhered to strips of Post-it^®^ labelling tape as a food source until used in the assays. The *O. insidiosus* were kept in a screened cage and given strips of *E. kuehniella* and pollen, as well as a bean plant (*Phaseolus vulgaris* ‘California Red Kidney’) between receipt and the start of the assay.

#### 2.1.3. Nematodes

Entomopathogenic nematode species, *S. feltiae*, *S. carpocapsae*, and *H. bacteriophora* were obtained from Sierra Biological (Lyndonville, NY, USA) and stored in a refrigerator at 5–6 °C until use for a maximum of 1 week. For every assay, one packet of 1 million nematodes was hydrated in a beaker with 500 mL room temperature (20–24 °C) tap water for 10 min. To verify viability and determine concentration of live infective juveniles (IJ) in the solution, a 50 µL sample was pipetted onto a petri dish and the number of live and dead nematodes were counted using a microscope at 40× magnification based on the methodology described in Kaya and Lacey 2007 [42]. This process was repeated 3 times and used to calculate the average concentration of live nematodes per mL. Sufficient water was added to dilute the nematode solution to equal 2000 live IJ/mL.

#### 2.1.4. Microbial Biopesticide

Botanigard^®^ 22WP (*B. bassiana* strain GHA) was tested as it is the most commonly used by growers in Ontario for thrips control (S. J., personal observation). Prior to use, viability of the product and enumeration of conidia in suspensions were assessed based on the methodology described in Inglis et al., 2012 [43].

### 2.2. Experimental Set Ups

#### 2.2.1. General Experimental Design

All experiments were set up as a complete block design (CBD), as the experimental arenas in all cases were small, and therefore placed close together inside a single growth chamber. In almost all cases, time was used as the blocking factor, repeating the experiments 2–7 times, to produce 15–43 replicates/treatment for each experiment. The exception to this was the choice trials involving *O. insidiosus* (see Section 2.2.3), where no blocking factor was used to achieve >20 replicates per treatment. See tables and figures in the Results section for the exact number of time blocks and total replicates (*N*) for each experiment.

#### 2.2.2. Predation Rate and Prey Preference of Predatory Mites

The experimental arena used for predatory mite assays was a modified Munger cell-type arena [44,45] consisting of two glass microscope slides, a 3 cm bean leaf disc, and craft foam with a 2 cm circle cut in the center that formed the arena space. After placing thrips larvae and mites inside, the cell was held firmly together with two binder clips, one on each side of the arena space.

Adult female predatory mites of unknown age were transferred into petri dishes with moistened filter paper for 24 h to starve them prior to all assays. First, all four mite species (*A. swirskii*, *I. degenerans*, *N. cucumeris*, *A. limonicus*) were tested in no-choice assays to determine the predation rate on first instar larvae of either OT or WFT over a 24 h period. As these mites are known to consume 4 to 7 first instar thrips larvae per day [35,46,47,48], we provided 8 larvae to ensure an excess of prey. Eight first instar larvae of either OT or WFT, and one predatory mite were moved into the arenas using a paint brush; arenas containing only thrips larvae were the control. Sealed arenas were placed in a growth chamber (25 °C, 70%RH, 16 h light) and the numbers of live OT, WFT, and mites were recorded after 24 h. Both fully consumed and intact thrips carcasses were counted as dead. This trial was repeated with second instar larvae for *A. swirskii*, as this mite is known to consume second instar, as well as first instar, larvae [49,50]. *Amblydromalus limonicus* can also consume second instar larvae but was omitted due to time constraints. As second instar thrips are larger, only 6 larvae were provided in each arena. Otherwise, the methodology was as described above.

For choice assays, *A. swirskii* and *N. cucumeris* (the two most common mites used by Ontario growers, S. Jandricic, personal observation) were provided with four OT and four WFT first instar larvae in a choice assay. Control arenas had no mites. Sealed arenas were placed in a growth chamber as above. After 8 h, the predatory mite was removed. The number of live thrips larvae for each species was recorded, then the arenas were re-sealed and returned to the growth chamber. The larvae were counted again 48 h later to verify species identifications of survivors as distinguishing features are more readily visible on second instar larvae (for description of differentiating features of larval OT and WFT, see [6,51]).

#### 2.2.3. Predation Rate and Prey Preference of *Orius insidiosus*

As with the predatory mites, the *O. insidiosus* assay was repeated comparing single-species (no-choice assay) and mixed-species (choice assay) dishes to determine prey preference, including control dishes in which no predator was added. The assay arena consisted of a bean leaf disc (47 mm) inside a small petri dish with an interior diameter of 47 mm. The leaf disc was sealed to the inner edge of the petri dish with a thin ring of plasticine to prevent thrips escape. Insects were transferred through a 4 mm diameter hole in the top of the dish just large enough to fit the tip of an aspirator. Once the insects were added, the hole was sealed with a small ball of plasticine.

For the no-choice assay, one adult *O. insidiosus* was added to the dish 24 h before the assay and starved of any protein sources (prey or pollen), and the dishes were kept in a growth chamber (25 °C, 70%RH, 16 h light). Twenty adult thrips (either OT or WFT) were placed in the dish and returned to the growth chamber. The number of surviving and dead adult thrips were counted after 24 h. For the choice assay, 16 adult thrips (8 OT and 8 WFT) were added to the dish and counted after 8 h.

#### 2.2.4. Efficacy of Entomopathogenic Nematodes

The arena used for the EPN laboratory assays follows the methods used in Saito and Brownbridge 2016 [52]. A 120 mL translucent plastic jar with a screw-on lid (Qorpak^®^, Berlin Packaging, Chicago, IL, USA) had a 4 cm hole cut in the lid to permit ventilation. A 5.5 cm diameter sticky card disc was cut to cover the opening of the jar. Five 1 mm air-holes were punched in the center of the card which was placed with the glue side facing the inside of the jar to catch emerging adult thrips during the assay. The opposite side of the sticky card disc was covered with wax paper. Fine mesh (200 μm) fabric was placed over the sticky card disc to catch any thrips that escaped through the air-holes. The lid was then screwed on, securing the sticky card disc and mesh firmly in place.

Moist potting mix (Agro-Mix^®^ G6, Fafard, Saint-Bonaventure, QC, Canada) was added to the bottom of the jars as a pupation substrate for the thrips. The potting mix is composed of 82% peat moss, 10% perlite, and 8% coconut husk fiber. Moisture content of the mix was standardized by adding 250 mL of water to 100 g (ca. 1 L) of oven-dried potting mix. After allowing time for the potting mix to hydrate, 13 g of mix was added to each cup and patted down gently so that the depth of the substrate was 2 cm. This depth was chosen as it has been found that most WFT and OT pupate within the upper 2 cm of substrate [53,54].

To obtain same-aged cohorts of pupae for assays, first instar WFT and OT (≤24 h difference in age) from miniature cucumber colonies [41] were moved onto detached chrysanthemum leaves (*Chrysanthemum indicum* cv. ‘Springdale Purple’, Syngenta Flowers North America, Gilroy, CA, USA) in 250 mL plastic containers (SOLO^®^, Dart Container Corporation, Mason, MI, USA). The containers were placed in a growth chamber (25 °C, 70%RH, 16 h photoperiod) for 6 days. Ten late second instar larvae nearing pupation were then moved onto a small piece of chrysanthemum leaf (approximately 2 cm^2^) on the surface of the substrate in the assay arena. The arenas were placed in the growth chamber (conditions as above). After three days, leaves were removed and inspected for larvae that did not pupate or died in the transfer. The number of dead or non-pupating larvae (3.3%) was recorded and subtracted from the starting number.

Then 1 mL of either nematode solution or water (control treatment) was pipetted evenly onto the potting mix. The surface area was 19.63 cm^2^, therefore the nematode application rate was equivalent to approximately 100 IJ/cm^2^, which is the highest recommended rate for ornamental crops given by nematode suppliers [55,56,57]. Sealed arenas were returned to the growth chamber. The combined duration of pre-pupa and pupa stages at 25 °C is 3.52 and 3.75 days for OT and WFT respectively [58]. Counts were conducted after 7 days to ensure all survivors had sufficient time to complete development. Sticky cards on the lids, the sides of the cup and potting mix surface were examined to determine the number of thrips that successfully reached adulthood.

Initially, only *S. feltiae* was tested against an untreated control, followed by simultaneous tests of *S. carpocapsae* and *H. bacteriophora* and untreated controls in a separate set of trials.

#### 2.2.5. Efficacy of *B. bassiana* GHA

The arena used for entomopathogenic fungi follows the “leaf disc sandwich” method described in Ugine et al., 2005 [59]. The “leaf disc sandwich” was created by stacking a 2.5 cm filter paper (Grade 417, VWR International, Mississauga, ON), two 2 cm diameter chrysanthemum leaf discs (‘Springdale purple’) with the abaxial sides facing each other, and a second filter paper on top. Prior to assembly, the abaxial surfaces of both leaves within the sandwich were sprayed with their respective treatments using a Potter spray tower and allowed to dry. Each filter paper was moistened with 35 µL of reverse osmosis (RO) water, and the sandwich was then put into a 30 mL translucent plastic portion cup (SOLO^®^) and sealed with a lid.

For the assays, 5 ml of solution was sprayed onto a 100 mm petri dish containing 6 leaf discs. Two discs the same diameter as the leaf discs (2 cm) cut from stiff plastic film were also included in each dish to confirm the conidia concentration applied to the leaf discs. The plastic discs were submerged in 0.01% surfactant (Triton^TM^ X-100, Dow Chemical Canada ULC, Calgary, AB, Canada) solution (2 to 5 mL depending on spray concentration) and vortexed to dislodge conidia. The number of conidia in the surfactant solution was determined using haemocytometer counts. The average number of conidia per mm^2^ applied to the leaf discs per rate are reported in Results.

Three rates of *B. bassiana* were applied to the discs: (1) 2.5 g/L—the maximum recommended label rate; (2) 1.25 g/L—the minimum recommended label rate; and (3) 0.125 g/L—10% of the minimum recommended label rate, as well as a water-only control. After the leaf disc sandwich had been assembled, ten 3-day old second instar larvae (either OT or WFT) were transferred to the top filter paper using a fine tip paintbrush. As per Ugine et al. (2005) [59], the thrips then crawled between the leaf discs for feeding, thereby exposing them to the EPF. The cups were then sealed and placed in a growth chamber (25 °C, 70%RH, 16 h photoperiod) for 5 days. The leaf discs, filter papers, and inside of the cup were then searched, and the number of dead and living larvae counted.

### 2.3. Statistical Analysis

Data were analyzed using Proc GLIMMIX in SAS^®^ Studio Version 3.8 (Copyright © 2013–2018, SAS Institute Inc., Cary, NC, USA). For all assays, to reach a minimum of 15 replicates, experiments were repeated over multiple time blocks. These experimental blocks were included as a random variable in analyses (total N for replicates and number of time blocks for each trial are reported in results tables). For all predator trials, data from dishes in which the predator died were omitted. In all cases, a generalized linear mixed model (GLMM) was used. The specific distribution type varied across assays as to best fit assumptions of variance, as did the use of comparison tests. Details are given below.

#### 2.3.1. Tests with Predatory Mites

In both the no-choice and choice assays, the difference in control mortality between thrips species was not statistically significant. Therefore, uncorrected mortality was analyzed with the control included in the model as a treatment. Predation rate (i.e., the total number of dead thrips, whether consumed entirely or killed) was analyzed using a Poisson distribution for no-choice assays. Contrast statements were conducted to determine differences between predation rates on OT and WFT within each predator species. Simple effect comparisons were used to determine the difference in predation rate between predator species for OT and WFT separately. For the choice assays, differences in the predation rate between OT and WFT used a Gaussian distribution, with contrast statements used to determine differences between the numbers of thrips species consumed for each predator.

#### 2.3.2. Trials with *O. insidiosus*

In both the *O. insidiosus* no-choice and choice assays, OT had higher mortality than WFT in the controls. Therefore, the corrected mortality rate was calculated using Abbott’s formula [60]. Differences in corrected mortality between thrips species were analyzed using a GLMM with a beta distribution.

#### 2.3.3. Trials Using Entomopathogens

For both the EPN and *B. bassiana* assays, the corrected thrips mortality was calculated using Abbott’s formula [60] with control mortality for each thrips species within each experimental block. The corrected mortality was analyzed using a beta distribution. For the *B. bassiana* trials, the effect of thrips species and *B. bassiana* rate on the corrected mortality of thrips was analyzed as in the *O. insidiosus* trials. As *S. feltiae* was tested in a separate trial from the other 2 species, each nematode species was analyzed separately to determine differences in mortality between OT and WFT, and differences between nematode species were not analyzed.

## 3. Results

### 3.1. Predation Rate and Prey Preference of Predatory Mites

In no-choice trials, there were significant differences between predator treatments in the number of first instar larvae killed or consumed (F_(4,204)_ = 39.7, *p* < 0.0001). There was no effect of thrips species (F_(1,204)_ = 2.71, *p* = 0.1014) or interaction (F_(4,204)_ = 0.57, *p* = 0.6877). *Amblyseius swirskii* consumed the most OT, which was significantly more than *I. degenerans* and *N. cucumeris*, but there was no significant difference between any predatory mite species with WFT as prey (Table 1). In paired contrasts between OT and WFT within each predator species, *A. swirskii* consumed significantly more OT first instar larvae than WFT larvae, whereas the other predators, *N. cucumeris*, *I. degenerans*, and *A. limonicus*, consumed equal numbers of both thrips species (Table 1).

In the no-choice trial with second instar thrips, there was a significant interaction between predator treatment and thrips species (F_(1,79)_ = 4.99, *p* = 0.0283), as well as predator treatment (F_(1,79)_ = 56.11, *p* < 0.0001) and thrips species (F_(1,79)_ = 4.99, *p* = 0.0283). Contrary to the results of the no-choice trials with first instar larvae, *A. swirskii* consumed 77% more second instar WFT than OT (Table 1).

In the 8 h choice trial, when presented with equal numbers of OT and WFT first instar larvae, there was a significant effect of thrips species (F_(1,17.17)_ = 32.29, *p* < 0.0001) but not predator treatment (F_(1,4.532)_ = 0.09, *p* = 0.7804) on predation rate, with no significant interaction (F_(1,17.17)_ = 0.10, *p* = 0.7597). Both predators appear to prefer OT over WFT. *Amblyseius swirskii* consumed 42% more OT than WFT, and *N. cucumeris* consumed 45% more OT than WFT (Table 1).

### 3.2. Predation Rate and Prey Preference of Orius insidiosus

In the no-choice trials, analysis of the corrected mortality found that *O. insidiosus* consumed twice as many OT adults compared to WFT (F_(1,38)_ = 21.10, *p* < 0.0001). However, when presented with both species in the choice trial, the effect of species was not significant (F_(1,34)_ = 1.81, *p* = 0.1879) (Table 2). For clarity, the non-corrected mortality rates are also shown in Table 2.

### 3.3. Efficacy of Entomopathogenic Nematodes

**Figure 1:**
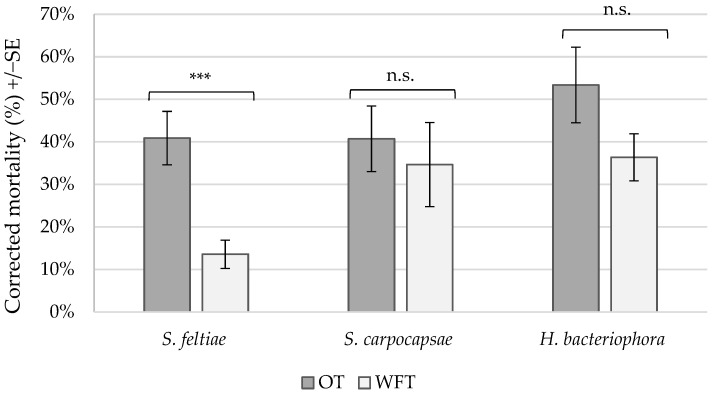
Mean corrected mortality (%) of pupating *Thrips tabaci* (OT) and *Frankliniella occidentalis* (WFT) when treated with entomopathogenic nematodes *Steinernema feltiae* (*N* = 20), *S. carpocapsae* (*N* = 15), or *Heterorhabditis bacteriophora* (*N* = 15) in laboratory assays. Replicates were conducted over 2 time blocks for each nematode species. *** indicates a significant difference between thrips species within nematode species > 0.001; ‘n.s.’ indicates no significant effect. Mortality was corrected using Abbott’s formula [60] using control mortality for each species within each experimental block.

Mortality rates in the untreated control treatments were high (47.0% and 45.7% for OT and WFT, respectively), but there was no significant difference in control mortality between thrips species (F_(1,57)_ = 0.09, *p* = 0.7684). There was no difference in corrected mortality between OT and WFT for *S. carpocapsae* (F_(1,27)_ = 0.23, *p* = 0.6361) nor *H. bacteriophora* (F_(1,27)_ = 2.57, *p* = 0.1205), which caused average corrected mortality rates of 37.6% (±8.73) and 44.9% (±7.62), respectively. There was, however, a significant difference between thrips species for *S. feltiae* (F_(1,37)_ = 14.32, *p* = 0.0005). For this species, the corrected mortality for OT (40.9% ± 6.27) was two times higher than that for WFT (13.6% ± 5.54) (Figure 1).

### 3.4. Efficacy of Entomopathogenic Fungi

In trials with the EPF, there was a significant effect of the rate of *B. bassiana* on thrips mortality (F_(2,92)_ = 16.49, *p* < 0.0001), but no effect of thrips species (F_(1,92)_ = 0.01, *p* = 0.9376) or interaction between rate and species (F_(2,92)_ = 0.05, *p* = 0.9509). There was no significant difference between high (2.5 g/L) and low (1.25 g/L) label rates on the corrected mortality of thrips (t_92_ = 1.52, *p* = 0.2886). The rate ten-fold lower than the low label rate (0.125 g/L) still caused 26% corrected mortality. However, this was significantly lower than both the high and low label rates (t_92_ ≥ 5.60, *p* ≤ 0.0002 for both) (Table 3).

## 4. Discussion

The primary goal of this study was to determine if biocontrol agents commonly used in floriculture biocontrol programs differed in their ability to control both WFT, a key pest, along with the novel greenhouse pest, OT. This research fills an important gap in the literature, as direct comparisons of biocontrol agents against OT and WFT were lacking, despite their coexistence in floriculture [6,61,62] and vegetable greenhouses [63,64]. In most cases, predators, entomopathogenic fungi (EPF), and entomopathogenic nematodes (EPN) performed equally well against OT as WFT, if not better.

An issue encountered in our screening of biocontrol agents against OT was the high control mortality in our EPN trial when compared to the mortality rates seen in the predatory mite assays (8.3%) and *B. bassiana* assay (14.5%). This difference may be due to a combination of the developmental stage used (pupae versus larvae), the assay environment, and duration of the assay. However, a relatively high control mortality is not unusual in laboratory trials of thrips pupation in soilless media. Saito and Brownbridge [52], upon whose assay method this trial was based, recorded a control mortality of WFT ranging from 29.2 to 38.2%. Helyer et al. [54] reported a control mortality of WFT of 31.3% in peat/loam substrate. Both the type of substrate and pupation depth may impact the natural mortality rate. Comparing substrate types, Ansari et al. [65] found the lowest control mortality in bark (12%), moderate mortality in peat (25.5%), and higher mortality in coir (31.5%). They posited that higher mortality in peat and coir might be attributed to suffocation in the relatively more compact and waterlogged peat and coir substrate. This was corroborated by Ebssa et al. [66], who found increasing mortality of WFT with increasing moisture content in peat-based potting mix, from only 6.1% mortality in the driest treatment up to 34.5% in the wettest treatment. Highly saturated peat is representative of typical conditions in commercial floriculture operations, and EPNs are typically applied using a soil drench with large volumes of water to promote survival and movement of the nematodes [67,68]. Although such high control mortality rates are not considered ideal for laboratory trials, it may be that this is an accurate representation of natural thrips survival in this cropping system.

Despite being the most commonly used species, the results of this study indicate that *S. feltiae* is less effective at infecting and killing WFT than OT, and that it is less effective for WFT than *S. carpocapsae* and *H. bacteriophora*. Saito and Brownbridge [52] reported a similarly low corrected mortality rate of 13.2% for WFT exposed to *S. feltiae* in potting mix substrate. Among the many published studies, there is no clear consensus as to which EPN species offers the best control of WFT or OT. There is research demonstrating the superiority of *H. bacteriophora* [25,27], *S. feltiae* [27,40], and *S*. *carpocapsae* [39,40], as well as studies that show the species are equally effective [20,69,70]. The lack of consensus in the literature may be due to the variety of assay conditions, species strains, and nematode culturing methods used. The documented differences in virulence between strains [25,26,27] make it difficult to generalize about the effectiveness of a species, or discern whether differences may be due to strain or assay method. Several studies demonstrate that substrate type impacts EPN efficacy. For example, *S. carpocapsae* is more mobile in clay, while *H. bacteriophora* performs well in peat-based potting mixes and other substrates with a high percentage of organic matter [71,72,73,74]. This highlights the importance of validating EPN results using commercial strains in the substrates in which they will be used, to get a clearer picture of how they will perform in real-life settings.

Our study was designed to rapidly screen a large number of BCAs to determine which may differ in their efficacy between OT and WFT, to potentially identify where current biocontrol programs may be failing, and to point towards future research questions. As such, our predator assays were of short duration. Previous research has demonstrated that over longer-term trials, satiation impacts the consumption rate of predatory mites, and that the effect of satiation differs between species [75]. Therefore, these results may not accurately reflect which predatory mite species can consume the most OT over its lifetime. Further, as WFT are larger than OT, the effect of satiation could exacerbate differences between thrips species, as predators would reach satiety faster when consuming WFT. Our trial also used adults collected at random from those received in biocontrol shipments, rather than creating single-aged cohorts. As predator age can impact consumption rate [18,76], using mites of unknown age may have introduced more variation in predation between individuals within each species. However, as all ages of predators are present in a greenhouse environment, this may offer a more realistic view of predation capacity, rather than maximum capacity, as would be the case if only the most voracious life stage were used.

Although all biocontrol agents performed well against OT in our lab trials (often better than on WFT), this does not reflect the experience of growers who struggle to control this species. It is not uncommon for researchers to find inconsistency between laboratory and field trial results. For example, *A. swirskii* was found to be a better predator of *Scirtothrips dorsalis* than *N. cucumeris* in greenhouse trials despite performing equally well in laboratory trials [77], and *A. limonicus* dramatically outperformed *N. cucumeris* as a WFT predator in greenhouse trials [34] despite having a very similar predation rate in laboratory trials [35]. In other studies, the efficacy of EPNs in greenhouse trials dropped substantially compared to laboratory assays for both WFT [69] and OT [20]. Many factors may contribute to these discrepancies, including the behaviors of both predator and prey, plant structure, and environmental factors. There are limitations inherent in laboratory trials that may explain why the BCAs included in this study fail to realize their potential when they are used in commercial operations.

The arenas used in laboratory assays such as these offer little opportunity for thrips to hide, escape, or exhibit other behaviors that influence predation rate. Uiterwaal and DeLong [78] found that arena size was the most important determinant of predation rate in experiments using ladybird beetles on a variety of prey types. The frequency with which predators and prey interact (‘encounter rate’) is among the main determinants of predation rate [19], and small arenas used in laboratory trials ensure an artificially high encounter rate. In whole plant studies, the within-plant distribution of both predator and prey will affect how likely they are to encounter each other, and have previously explained failures in the biocontrol of novel greenhouse species [79]. Predator avoidance behavior can also reduce the encounter rate between predators and prey that would otherwise share the same habitat. Research has found that *O. insidiosus* consumes more WFT even when presented with other flower-feeding thrips species on pepper host plants [19,80]. Though *Frankliniella tritici* and *F. bispinosa* are equally acceptable food sources for *O. insidiosus* as WFT, these species retreat more readily in the presence of the predator, thus reducing their encounter rate relative to the slower WFT [19]. To date, study of OT defensive/avoidant behaviors in the presence of predators is limited [9], and research on how these behaviors compare to WFT may provide further clarity.

The within-plant distribution of different thrips species is also relevant to the efficacy of microbial insecticides, as the pest needs to contact viable spores to cause mortality. Differences in the movements of thrips within and between plants throughout the day could be of particular relevance to foliar applications of EPFs. Dispersal patterns of OT and WFT have been shown to vary throughout the day; however, the time of peak activity differs among reports [81,82,83]. Research from Israel found that OT were more active in the morning [82], whereas research conducted in New York state found that OT were more active in the afternoon [83]. The researchers posited that this difference may be due to the cooler overnight and morning temperatures in New York state compared to the warmer Mediterranean climate [83]. If OT in the greenhouse are less active in the afternoon, as they are in Israel, this could reduce their contact with foliar applications of EPFs which are typically applied later in the day in commercial greenhouses due to re-entry interval restrictions.

Differences in leaf surface and plant architecture make every plant species a unique landscape that can change the movement and behavior of both predator and prey, resulting in differences in predator efficacy between crops. The impact of host plants on thrips predator efficacy has previously been documented for *N. cucumeris* [36,47,84], *O. insidiosus* [84,85], and *A. swirskii* [11,86]. The host plant, as well as spray equipment and technique, can also impact the efficacy of EPFs [28,87,88]. The dense canopy structure of many floriculture crops makes adequate coverage challenging, resulting in high spray deposition on the top leaves and upper surface, and substantially less on the underside of leaves and canopy interior where pests are often found [88]. Predator efficacy can also be impacted by environmental conditions. The optimal temperatures of predators and prey species can create advantages for different species under different growing conditions. Research has found that *A. limonicus* can provide better control than other phytoseiid mites under cool, short-season conditions [89]. Given that OT populations persist in the greenhouse in late fall and winter [6], the potential to provide better control during cooler months makes *A. limonicus* worthy of further study.

Despite the limitations of laboratory trials, they are an essential first step in the process to determine efficacy. Investigations must begin in small, controlled, laboratory assays before scaling up to semi-field and field environments [90]. Our results establish a fundamental capacity for these BCAs to consume or kill OT as well as they do WFT, and they suggest areas for further study. Specifically, further investigation of *A. swirskii* as an effective predator is warranted, based on increased predation of this particular species of mite on OT first instars over WFT. To save money on biocontrol programs, ornamental growers in Ontario will often use the less expensive *N. cucumeris* over *A. swirskii*, as both can be effective for WFT. If, for OT, *A. swirskii* outperform *N. cucumeris* in the greenhouse to the same extent as these lab trials, this may explain some of the biocontrol failures seen at certain facilities in Ontario. Further, *O. insidiosus* is rarely used in potted ornamentals in Ontario (also due to cost), except in severe outbreak situations [91]. Given its high predation rate on adult WFT and OT, this predator may be a necessary addition to greenhouses battling regular OT outbreaks. Similarly, nematode species other than *S. feltiae* are rarely used in commercial greenhouses. The similar performance of *S. carpocapsae* and *H. bacteriophora* for OT (and superior performance against WFT) warrants further testing of these species in whole plant trials. More preliminary research is also needed on other commercially available microbial pesticides against OT. Although there were no differences between OT and WFT for BotaniGard WP, previous research has shown a wide variation in response to different species and strains of EPF when it comes to greenhouse pests in the same superfamily [92]. Other commercially available *Beauveria* strains, such as Velifer (*B. bassiana* strain PPRI 5339) or Bioceres (strain ANT-03), or Lalguard M52 (*M. brunneum* M52), and different formulations (e.g., powder vs. oil-based carriers) may prove more effective against OT and other non-WFT species both in the lab and in the greenhouse.

Lastly, more research into the ecology of OT as a pest of ornamentals is necessary to elucidate non-pesticide solutions for this pest. Biocontrol agents are living organisms that interact with each other in many ways that can affect pest control outcomes. Ecological factors such as within-plant distribution [93], intraguild predation [16,17], and interspecific competition [94] between thrips species can affect the realized efficacy of an IPM program. As living organisms, BCAs can also be harmed by pesticides, thus reducing their effectiveness in crops where pesticides are still being used [95,96,97,98]. Coupled with the continuation of biocontrol screening in research greenhouse trials, future research will need to address these ecological factors to develop a reliable IPM strategy that can be effective for both thrips species.

## Figures and Tables

**Table 1:** Mean predation rates (number of thrips killed or consumed) ± standard error (SE) of predatory mite species on *Thrips tabaci* (OT) and *Frankliniella occidentalis* (WFT) in 24 h no-choice trials and 8 h choice trials, and Tukey’s adjusted *p*-value of contrasts between thrips species. ‘Control’ represents natural mortality in no-predator treatments. Total N were collected over a series of time blocks (reported in sub-titles). Different letters represent significant differences (α = 0.05) between predator species within each thrips species for each trial as determined by simple effects comparisons, and asterisks (*) indicate significant differences between OT and WFT for each predator.

	OT			WFT			Adj. *p*-Value
Predator Treatment	*N*	Avg.		*N*	Avg.		(OT vs. WFT)
No-choice (24 h), first instar (7 blocks)							
*A. swirskii*	24	6.2 ± 0.36	A	22	4.5 ± 0.42	A	0.0139 *
*A. limonicus* ^1^	15	5.1 ± 0.65	AB	15	4.7 ± 0.56	A	0.6935
*I. degenerans* ^2^	20	4.1 ± 0.37	B	19	3.3 ± 0.33	A	0.1772
*N. cucumeris*	21	3.7 ± 0.44	B	20	3.3 ± 0.42	A	0.5364
Control	35	0.7 ± 0.12	C	29	0.7 ± 0.15	B	0.9728
No-choice (24 h), second instar (2 blocks)							
*A. swirskii*	21	1.0 ± 0.20		21	1.9 ± 0.28		0.0022 *
Control	21	0.1 ± 0.07		21	0.1 ± 0.1		1.0000
Choice (8 h), first instar (5 blocks)							
*A. swirskii*	20	2.7 ± 0.22		20	1.6 ± 0.24		<0.0001 *
*N. cucumeris*	22	2.8 ± 0.23		22	1.5 ± 0.18		<0.0001 *
Control	43	0.7 ± 0.13		43	0.4 ± 0.08		0.0813

^1^ *A. limonicus* was used in 3 of the 7 time blocks. ^2^ *I. degenerans* was used in 4 of the 7 time blocks.

**Table 2:** Predation rates (mean number (#) of thrips dead or consumed ± standard error) and corrected mortality of *Orius insidiosus* on adult *Thrips tabaci* (OT) and *Frankliniella occidentalis* (WFT) in 24 h no-choice trials and 8 h choice trials. ‘Control’ represents natural mortality in no-predator treatments. Different letters indicate significant difference between corrected mortality ^1^ rate (%) of OT and WFT (α = 0.05) within the *O. insidiosus* treatments. Total *N* were collected over a series of series of time blocks (reported in table headings).

	No Choice, 24 h (3 Blocks)			Choice, 8 h (1 Block)		
	*N*	OT	WFT	*N*	OT	WFT
Control, #dead	25	3.8 ± 0.5	0.8 ± 0.2	20	1.8 ± 0.36	0.8 ± 0.12
*O. insidiosus*, #dead	22	13.0 ± 1.3	5.3 ± 0.9	19	5.9 ± 0.54	3.9 ± 0.44
Corrected mortality ^1^, %		68.8 ± 8.1 A	27.2 ± 5.3 B		49.9 ± 6.6 ns	34.2 ± 4.7 ns

^1^ Mortality rate was corrected using Abbott’s formula [60] using the control mortality for each thrips species within each experimental block.

**Table 3:** Mean conidia density and corrected mortality rate ± standard error of *Thrips tabaci* (OT), *Frankliniella occidentalis* (WFT), and average mortality at three rates of *Beauveria bassiana* strain GHA (BotaniGard^®^ WP) in a laboratory assay. *N* = 17 (per species × rate), over 4 time blocks. Significant differences in average mortality between rates is indicated by different letters (α = 0.05).

Rate	Conidia/mm^2^	% OT Mortality ^1^	% WFT Mortality ^1^	Average % Mortality ^1^
2.5 g/L	1161.7	61.3 ± 6.63	59.9 ± 6.55	60.6 ± 4.59 A
1.25 g/L	604.6	50.1 ± 5.70	52.2 ± 6.50	51.1 ± 4.18 A
0.125 g/L	75.9	25.5 ± 3.46	25.8 ± 6.93	25.7 ± 3.81 B

^1^ Mortality rate was corrected using Abbott’s formula [60] using the control mortality for each thrips species within each experimental block.

## Data Availability

Data are available on request from corresponding author (ashley.summerfield@vinelandresearch.com).
